# Erlotinib for elderly patients with non-small-cell lung cancer: Subset analysis from a population-based observational study by the Ibaraki Thoracic Integrative (POSITIVE) Research Group

**DOI:** 10.3892/mco.2013.154

**Published:** 2013-07-23

**Authors:** KOICHI KURISHIMA, HIROAKI SATOH, TAKAYUKI KABURAGI, YOSHIHIRO NISHIMURA, YOKO SHINOHARA, MASAHARU INAGAKI, TAKEO ENDO, TAKEFUMI SAITO, KENJI HAYASHIHARA, NOBUYUKI HIZAWA, HIROYUKI NAKAMURA, TAKESHI NAWA, KATSUNORI KAGOHASHI, KOJI KISHI, HIROICHI ISHIKAWA, HIDEO ICHIMURA, TOSHIO HASHIMOTO, YUKIO SATO, MITSUAKI SAKAI, KOICHI KAMIYAMA, TAKESHI MATSUMURA, KOJI UNOURA, TOSHIHIKO FUKUOKA, KEIKO UCHIUMI, AKIHIRO NOMURA, KINYA FURUKAWA

**Affiliations:** 1 Tsukuba University Hospital, Tsukuba, Ibaraki 305-8576, Japan; 2 Mito Medical Center, University of Tsukuba, Mito, Ibaraki 310-0015, Japan; 3 Ibaraki Prefectural Central Hospital and Regional Cancer Center, Kasama, Ibaraki 309-1793, Japan; 4 Mito Chuo Hospital, Mito, Ibaraki 311-1135, Japan; 5 Tsuchiura Kyodo General Hospital and Regional Cancer Center, Tsuchiura, Ibaraki 300-0053, Japan; 6 National Hospital Organisation, Mito Medical Center Hospital, Mito, Ibaraki 311-3193, Japan; 7 Ibaraki Higashi National Hospital, Tokai, Ibaraki 319-1113, Japan; 8 Tokyo Medical University, Ibaraki Medical Center Hospital, Ami, Ibaraki 300-0395, Japan; 9 Hitachi General Hospital, Hitachi, Ibaraki 317-0077, Japan; 10 Tsukuba Medical Center Hospital and Regional Cancer Center, Tsukuba, Ibaraki 305-8558, Japan; 11 Mito Saiseikai General Hospital, Mito, Ibaraki 311-4198, Japan; 12 Tsukuba Memorial Hospital, Tsukuba, Ibaraki 300-2622, Japan; 13 Ibaraki Seinan Medical Center Hospital, Sakai, Osaka 306-0433, Japan; 14 JA Toride Medical Center, Toride, Ibaraki 302-0022, Japan

**Keywords:** erlotinib, elderly, non-small-cell lung cancer, observational study, population-based

## Abstract

The incidence and mortality of lung cancer have increased worldwide over the last decades, with an observed increased incidence particularly among elderly populations. It has not yet been determined whether erlotinib therapy exhibits the same efficacy and safety in elderly and younger patients with non-small-cell lung cancer (NSCLC). The aim of this retrospective subgroup analysis of data from a population-based observational study was to assess the efficacy and safety of erlotinib in an elderly (≥75 years, n=74) and a younger group of patients (<75 years, n=233) who received treatment for NSCLC. The time to treatment failure was similar in the elderly [median, 62 days; 95% confidence interval (95% CI): 44–80 days] compared with the younger group (median, 46 days; 95% CI: 35–53 days) (P=0.2475). The overall survival did not differ between the elderly and younger groups (median, 170 days; 95% CI: 142–239 days vs. median, 146 days; 95% CI: 114–185 days, respectively) (P=0.7642). The adverse events did not differ in incidence between the groups and were manageable, regardless of age. Among the NSCLC patients receiving erlotinib treatment, the outcomes of the elderly (≥75 years) and younger (<75 years) groups of patients were similar in our population-based observational study.

## Introduction

In developed countries, the life expectancy of the general population is on the increase, leading to an increased incidence of malignant diseases among elderly individuals. Among malignant diseases, the incidence and mortality of lung cancer has increased worldwide over the last decades (1,2). Due to the recent advances in the medical management of lung cancer, the development of new drugs such as epidermal growth factor receptor-tyrosine kinase inhibitors (EGFR-TKIs), the higher standards of medical care and the more widely available health services, survival of elderly patients may have been altered. Due to the increase in the incidence of lung cancer among elderly individuals, the efficacy and safety of EGFR-TKIs for the treatment of elderly patients with non-small-cell lung cancer (NSCLC) have been investigated in previous clinical trials (3–7), although those in clinical practice have not yet been evaluated. Therefore, additional studies are required, specifically focusing on EGFR-TKI efficacy and safety in a population-based evaluation in unselected patients.

Erlotinib, similar to gefitinib, is a reliable EGFR-TKI and has been prescribed for numerous NSCLC patients (8). In a previous phase III study (BR.21) that compared erlotinib with placebo in the second- or third-line treatment of NSCLC patients who were not responding to standard chemotherapy, erlotinib was confirmed to significantly prolong overall survival (OS), progression-free survival and the time to deterioration of lung cancer-related symptoms (cough, dyspnea and pain) as a quality of life measure (9). Successful results were also reported by a combined analysis of two phase II clinical studies (JO16565 and JO18396) conducted in Japan. The objective response and disease control rates were 28% [95% confidence interval (CI): 20.0–37.9%] and 49% (95% CI: 39.2–59.0%), respectively, whereas the time to progression was 10.7 weeks (95% CI: 8.1–18.3 weeks) and the OS was 13.8 months (95% CI: 11.4–18.1 months) (10). Erlotinib was demonstrated to be effective in EGFR mutation-positive patients (11,12), similar to gefitinib, although it was also suggested to be effective in EGFR mutation-negative patients (11,13).

A population-based observational study was recently conducted in the Ibaraki prefecture to investigate the usefulness of erlotinib in lung cancer treatment by collecting and analyzing data from all the patients receiving erlotinib, irrespective of their individual characteristics (14). In this subset analysis, we evaluated the association of age with the treatment results of erlotinib in patients with NSCLC, by comparing the outcomes between the elderly (≥75 years) and younger patients (<75 years) who were enrolled in this population-based observational study.

## Materials and methods

### Patients

Fourteen institutions (17 departments) located in the Ibaraki prefecture (area, 6,095 km^2^; population, ~3 million) participated in the present retrospective study, which included patients who were treated with erlotinib at these institutions between December, 2007 and December, 2010. In total, 307 patients were included in the study. Of these, 74 were aged ≥75 years (elderly group) and 233 were aged <75 years (younger group). All the patients demonstrated histological or cytological evidence of NSCLC. Histopathological diagnoses were defined according to the World Health Organization (WHO) classification system and the patients were staged according to the Union for International Cancer Control (UICC) tumor-node-metastasis (TNM) staging system.

The patient characteristics, efficacy and safety were evaluated using patient data extracted from the database of each institution. Tumor responses were classified as complete response (CR), partial response (PR), stable disease (SD), progressive disease or not evaluable, according to the response evaluation criteria in solid tumors (RECIST), version 1.1.

The present observational study conformed to the Ethical Guidelines for Clinical Studies issued by the Ministry of Health, Labor and Welfare of Japan.

In this subset analysis, patients were divided into two groups: those aged ≥75 years (elderly group) and those aged <75 years (younger group). Patient characteristics, efficacy and toxicity of erlotinib, time to treatment failure (TTF) and OS were compared between the two groups.

### Statistical analysis

Patient survival time was calculated from the date of erlotinib therapy initiation to the date of death or latest follow-up contact of the patient. The survival rate was analyzed by the Kaplan-Meier method and comparisons were performed using the log-rank test. P<0.05 was considered to indicate a statistically significant difference.

## Results

### Patient characteristics

During the study period, a total of 307 patients were treated with erlotinib and they were all evaluated for efficacy and safety in this analysis. Of the 307 patients, 74 were aged ≥75 years (elderly group) and 233 were aged <75 years (younger group). There were no clinically significant differences in baseline characteristics between the two age groups, apart from PS and treatment line of erlotinib (Table I).

### Efficacy

Tumor response was determined among patients according to age and treatment (Table II). Of the 74 elderly patients, 1 (1.4%) exhibited CR, 5 (6.8%) exhibited PR and 37 (50%) had SD. Of the 233 younger patients, 1.3, 10.7 and 30.5% had CR, PR and SD, respectively. The disease control rate (CR+PR+SD) was 58.2 and 42.5% in the elderly and younger groups, respectively, with a statistically significant difference (P=0.0228).

**Figure 1:**
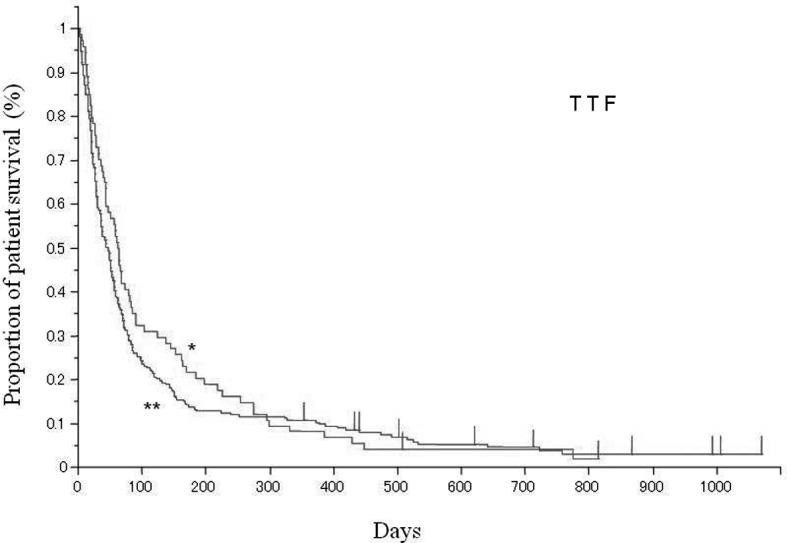
Time to treatment failure (TTF) in the ≥75 years (*) and in the <75 years (**) groups of patients.

**Figure 2:**
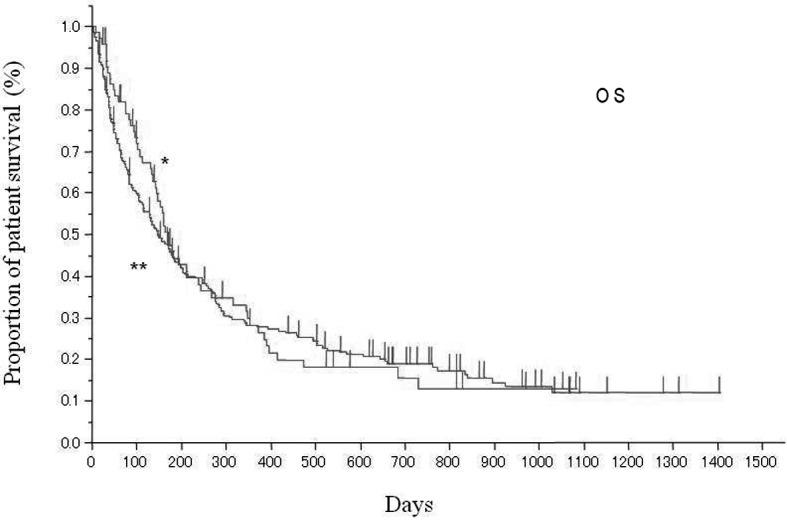
Overall survival (OS) in the ≥75 years (*) and in the <75 years (**) groups of patients.

TTF in the two age groups is shown in Fig. 1. The median TTF was 62 days (95% CI: 44–80 days) and 46 days (95% CI: 35–53 days) in the elderly and younger group, respectively. There was no statistical difference between the two groups (P=0.2475). The OS in the two age groups is shown in Fig. 2. The median OS was 170 days (95% CI: 142–239 days) and 148 days (95% CI: 114–185 days) in the elderly and the younger group, respectively. There was no statistical difference between the two groups (P=0.7642).

In the elderly group of patients, TTF was not different between EGFR mutation-positive and -negative patients (73 vs. 44 days, respectively; P=0.4585). No difference was observed in OS between the two types (268 vs. 160 days, respectively; P=0.5386).

### Toxicity

The incidence of adverse events >grade 3 in the two patient groups is presented in Table III. The elderly group exhibited a slightly higher incidence in hepatotoxicity, diarrhea and pulmonary toxicity, although the difference was not statistically significant. The younger group of patients exhibited a slightly higher incidence in skin rash, which was of no statistical significance.

### Efficacy and toxicity in patients aged ≥80 years

The differences between 20 patients aged ≥80 years and 287 aged <80 years were also analyzed. The disease control rate (CR+PR+SD) was 60.0% in the former and 45.3% in the latter group. There was no statistically significant difference between the two groups (P=0.2484). There was also no statistically significant difference in TTF and OS between the two groups (TTF, 67 vs. 49 days, respectively, P=0.6538; OS, 153 vs. 160 days, respectively, P=0.4956). There was no difference in the incidence of >grade 3 skin rash, hepatotoxicity, diarrhea or pulmonary toxicity between the two groups.

## Discussion

Prior to EGFR-TKI application in the treatment of elderly NSCLC patients, single-agent therapy with vinorelbine or docetaxel was the most frequently used systemic treatment option (15). Over the last few years, treatment of elderly NSCLC patients with EGFR-TKIs has attracted attention and has been investigated by previous studies (3–7). A phase II clinical trial conducted by Jackman *et al*(6) on chemotherapy-naive NSCLC patients aged ≥70 years, evaluated 80 patients and reported that the disease control rate was 51%, whereas time to progression and median survival time were 3.5 and 10.9 months, respectively (6). Inoue *et al*(5) prescribed gefitinib, another EGFR-TKI, for 30 patients with poor PS or those aged 75–79 years and reported that the disease control rate was 90%, whereas median progression-free survival and median survival time were 6.5 and 17.8 months, respectively. Their study group recently verified safety and efficacy of first-line gefitinib in 31 patients aged ≥75 years harboring EGFR mutations (7). Platania *et al*(4) reported their results from 43 patients aged ≥70 years. In their retrospective study, the disease control rate was 49% and the median progression-free survival and median survival time were 3 and 8.4 months, respectively (4). Recently, a phase II randomized trial conducted by Chen *et al*(3) investigated the use of erlotinib in 57 chemonaive patients aged ≥70 years and reported a response rate of 22.7%, with a median progression-free survival and median survival time of 4.57 and 11.67 months, respectively (3). Of note, that study also reported that EGFR mutation-positive patients exhibited better survival compared to those with EGFR wild-type disease (3).

Our POSITIVE study was a relatively large population-based observational study on 307 NSCLC patients and the results supported the use of erlotinib as a treatment for NSCLC (14). Elderly patients aged ≥75 years constituted 24.1% of the POSITIVE study population. The subgroup analysis of the POSITIVE population demonstrated that older and younger patients benefited equally from erlotinib treatment, without significant differences in TTF and OS. In the present subset analysis, the median TTF and OS in the elderly group of patients was 62 and 170 days, respectively. In the majority of the above-mentioned studies, the enrolled patients were aged ≥70 years and erlotinib was administered as first-line therapy (3–6). By contrast, the elderly patients in our study were unselected ordinary patients aged ≥75 years and 87.8% were administered erlotinib as second- or later-line therapy. This may be the reason for the OS among our elderly patients being worse compared to that reported by those previous studies. In addition, differences in the treatment following erlotinib therapy may affect OS. In this study, no unexpected adverse events attributable to advanced age were observed. Rash, diarrhea, hepatotoxicity and pulmonary toxicity were the most common toxicities and they were mostly manageable. Pulmonary toxicity was of no particular concern in the elderly patients, although grade 3 or higher pulmonary toxicity was observed in 4 patients (5.4%) in the elderly and 7 (3.0%) in the younger group. Of note, the disease control rate (CR+PR+SD) was higher in the elderly compared to the younger group of patients. Since over half of the patients included in this study had an unknown EGFR mutation status, the results may be attributable to the characteristics of the EGFR mutation in this study population. In this retrospective subgroup analysis of POSITIVE data, TTF and OS did not differ between the two age groups and the disease control rate was approximately equal in the two groups. The treatment efficacy was almost the same as that previously reported (3–7), although our study involved unselected ordinary patients. There was no notable difference in the frequency or severity of erlotinib-related toxicity between the two groups. Therefore, erlotinib treatment may be effective against NSCLC and tolerable in clinical practice, regardless of age. In addition, the efficacy and safety of erlotinib in our patients aged ≥80 and in those aged ≥75 years were approximately the same.

This study had several limitations. This was a retrospective subset analysis of a population-based observational study. Therefore, it was not designed to assess statistically significant differences in treatment effects between the elderly and younger subgroups. The limited sample size and imbalance of the size between the two study populations may influence the results. Approximately half of the patients from each age group had an unknown EGFR mutation status. The treatment lines were different between the two age groups, which may also affect the results. However, the data presented in this study may contribute important information and key study data on the elderly and younger subgroups receiving erlotinib therapy. These analyses provide informative results that may assist clinicians in selecting the appropriate targeted therapy and may also provide guidance in the design of trials involving targeted therapies for the elderly NSCLC patients.

The efficacy of erlotinib was maintained in patients aged ≥75 years. This result, combined with an acceptable toxicity profile in both the elderly and the younger patient groups, supports the use of erlotinib as a treatment for advanced NSCLC in all age groups.

## Figures and Tables

**Table I:** Characteristics of the elderly and younger groups of patients.

Characteristics	Patients ≥75 years (elderly group)	Patients <75 years (younger group)
Total no. of patients	74	233
Gender (male/female)	50/24	152/81
Performance status		
0–1/2/3–4	49/21/4	158/42/33
Pathology		
AD/SQ/other	53/9/12	180/33/20
Smoking		
Never/smoker/unknown	30/42/2	76/152/5
Treatment line		
1st/2nd/3rd/4th or later	9/21/18/26	11/50/58/114
EGFR mutation status		
Positive/negative/unknown	9/23/42	46/62/125

AD, adenocarcinoma; SQ, squamous cell carcinoma; EGFR, epidermal growth factor receptor.

**Table II:** Tumor response in patients aged ≥75 and <75 years.

Response (%)	Patients ≥75 years (elderly group)	Patients <75 years (younger group)
Complete response	1 (1.4)	3 (1.3)
Partial response	5 (6.8)	25 (10.7)
Stable disease	37 (50.0)	71 (30.5)
Progressive disease	26 (35.1)	103 (44.2)
Not evaluable	5 (6.8)	31 (13.3)
Response rate	6 (8.2)	28 (12.0)
Disease control rate	43 (58.2)	99 (42.5)

**Table III:** Toxicity >grade 3 in the elderly and younger groups of patients.

Toxicity (%)	Patients ≥75 years (elderly group)	Patients <75 years (younger group)
Skin rash	2 (2.7)	21 (9.0)
Hepatotoxicity	3 (4.1)	2 (0.9)
Diarrhea	1 (1.4)	1 (0.4)
Pulmonary toxicity	4 (5.4)	7 (3.0)

## References

[1] Bilello KS, Murin S, Matthay RA (2002). Epidemiology, etiology, and prevention of lung cancer. Clin Chest Med.

[2] Gabrielson E (2006). Worldwide trends in lung cancer pathology. Respirology.

[3] Chen YM, Tsai CM, Fan WC (2012). Phase II randomized trial of erlotinib or vinorelbine in chemonaive, advanced, non-small cell lung cancer patients aged 70 years or older. J Thorac Oncol.

[4] Platania M, Agustoni F, Formisano B (2011). Clinical retrospective analysis of erlotinib in the treatment of elderly patients with advanced non-small cell lung cancer. Target Oncol.

[5] Inoue A, Kobayashi K, Usui K, North East Japan Gefitinib Study Group (2009). First-line gefitinib for patients with advanced non-small-cell lung cancer harboring epidermal growth factor receptor mutations without indication for chemotherapy. J Clin Oncol.

[6] Jackman DM, Yeap BY, Lindeman NI (2007). Phase II clinical trial of chemotherapy-naive patients > or=70 years of age treated with erlotinib for advanced non-small-cell lung cancer. J Clin Oncol.

[7] Maemondo M, Minegishi Y, Inoue A (2012). First-line gefitinib in patients aged 75 or older with advanced non-small cell lung cancer harboring epidermal growth factor receptor mutations: NEJ 003 study. J Thorac Oncol.

[8] Reck M, Mok T, Wolf J (2011). Reviewing the safety of erlotinib in non-small cell lung cancer. Expert Opin Drug Saf.

[9] Bezjak A, Tu D, Seymour L, National Cancer Institute of Canada Clinical Trials Group Study BR.21 (2006). Symptom improvement in lung cancer patients treated with erlotinib: quality of life analysis of the National Cancer Institute of Canada Clinical Trials Group Study BR.21. J Clin Oncol.

[10] Kubota K, Nishiwaki Y, Tamura T (2008). Efficacy and safety of erlotinib as monotherapy for Japanese patients with advanced non-small-cell lung cancer: a phase II study. J Thoracic Oncol.

[11] Pirker R, Su W, Rooneem R (2008). Clinical outcome with erlotinib in relation to biomarker status: analysis from the open-label TRUST study in advanced non-small-cell lung cancer (NSCLC). Ann Oncol.

[12] Massuti B, Moran T, Porta R (2009). Multicenter prospective trial of customized erlotinib for advanced non-small-cell lung cancer (NSCLC) patients (p) with epidermal growth factor receptor (EGFR) mutations: Final results of the Spanish Lung Cancer Group (SLCG) trial. J Clin Oncol.

[13] Cappuzzo F, Ciuleanu T, Stelmakh L (2009). SATURN: A double-blind, randomized, phase III study of maintenance erlotinib vs. placebo following nonprogression with first-line platinum-based chemotherapy in patients with advanced NSCLC. J Clin Oncol.

[14] Kaburagi T, Satoh H, Hayashihara K (2013). Observational study on efficacy and safety of erlotinib in patients with non-small-cell lung cancer in Ibaraki, Japan. Oncol Lett.

[15] Ardizzoni A, Tiseo M (2004). Second-line chemotherapy in the treatment of advanced non-small cell lung cancer (NSCLC). J Chemother.

